# Effects of prescribed medical cannabis and alcohol on real-world driving performance (CAN-TRACK): a study protocol for a two-phase trial

**DOI:** 10.1186/s13063-026-09512-x

**Published:** 2026-02-06

**Authors:** Thomas R. Arkell, Amie C. Hayley, Blair Aitken, Xinyun Hu, Brooke Manning, Luke A. Downey

**Affiliations:** Centre for Mental Health and Brain Sciences, Swinburne University of Technology, Melbourne, VIC Australia

**Keywords:** Medical cannabis, Cannabis, THC, CBD, Alcohol, Driving, Cognition

## Abstract

**Background:**

Medical cannabis is now commonly prescribed for a range of chronic health conditions. Many medical cannabis products contain delta-9-tetrahydrocannabinol (THC), the intoxicating component in cannabis, though it is unclear whether these products produce impairment when used as prescribed and at therapeutic doses. With current Australian laws prohibiting driving with any amount of THC in one’s system, this trial aims to generate novel data on the impact of prescribed medical cannabis on real-world driving performance to inform road safety policy.

**Methods:**

This is a two-phase trial, with the first phase being a semi-naturalistic cohort study involving 72 patients with physician-diagnosed chronic pain, anxiety, or insomnia (*n* = 24 per group) who will complete repeated on-track driving assessments before and after consuming a standard dose of their medical cannabis prescription. The second phase is a randomised, placebo-controlled, double-blind, and crossover study involving 24 healthy participants who will complete the same repeated on-track driving assessments before and after consuming either alcohol (0.05% blood alcohol concentration) or placebo. Participants in both phases will also complete repeated cognitive assessments and assessments of subjective state and provide biological samples for analysis of cannabinoid (phase 1) and alcohol (phase 2) concentrations. The primary outcome measure is lateral vehicular control. Data will be analysed using a series of mixed-effect and generalised linear mixed models to assess changes in outcome measures over time.

**Trial registration numbers:**

ACTRN 17/09/2024 for 12624001118594 and 24/09/2024 for 12624001163594.

**Supplementary Information:**

The online version contains supplementary material available at 10.1186/s13063-026-09512-x.

## Introduction

### Background and rationale {6a}

Medical cannabis has been legal in Australia since November 2016. At the time of writing, Australia’s federal medicine and therapeutic regulatory agency—the Therapeutic Goods Administration (TGA)—had granted > 3.5 million approvals for access to medical cannabis. Of the >860,000 approvals for which product type and health indication data were available, approximately 80% were for Schedule 8 (S8) products containing THC. The remainder were for Schedule 4 (S4) products where cannabidiol (CBD) comprises > 98% of the total cannabinoid content. Nearly 50% of these approvals were for the treatment of chronic pain, with anxiety and sleep disorders (principally insomnia) the next most common indications for prescribing [1].

The intoxicating effects of THC have been extensively documented in laboratory investigations (e.g. [2–5]). When examining the magnitude and duration of drug effects on driving, ‘impairment’ is widely operationalised using the *standard deviation of lateral position* (SDLP), a measure of lane weaving and a proxy measure for general vehicular control. A recent meta-analysis concluded that THC-induced impairment in driving performance typically lasts for 3–8 h depending on the dose, route of administration, and frequency of cannabis use [6]. It is important to note that inhaled and oral cannabinoid products present unique pharmacokinetic profiles, which in turn produce variations in actual and perceived psychoactive drug effects, primarily due to differences in absorption, peak, and metabolic distribution [7, 8]. Inhaled cannabinoid products have a rapid onset of action and a relatively short window of peak effects, while oral products have a slower onset and longer duration of action [9].

Although prior acute drug administration research has established that cannabis can impair driving [10], the application of these findings to patients who are receiving long-term, stable treatment with medical cannabis is uncertain [11]. This is because previous research has primarily focused on investigating the effects of intentionally intoxicating doses of THC in healthy volunteers [6]. Under the Australian medical cannabis regulatory framework, patients are given a prescription for a specific cannabinoid product, much like how pharmaceutical medications are prescribed with pharmacy labels explicitly detailing dosage and frequency of use. This is a marked contrast to recreational cannabis, which is typically consumed via smoking and is often of unknown strength and quality. This key difference underscores the need for research that focuses on providing safety-relevant data specifically tailored to patient populations who are using medical cannabis as prescribed by a healthcare professional.

### Objectives {7}

The primary aim of this trial is to determine if and to what extent prescribed medical cannabis impacts on-track driving performance. This trial will also examine the effects of prescribed medical cannabis on cognitive function, subjective drug effects, and perceived driving ability. To provide a comparison to these findings, a secondary randomised, placebo-controlled trial will assess how a threshold legal dose of alcohol (0.05% blood alcohol concentration) impacts each of the same measures employed in phase 1. As BACs of ≤ 0.049% are legally permitted for drivers in Australia and numerous other countries, findings from phase 2 will provide an ecologically valid point of reference to findings obtained in phase 1.

### Trial design {8} and choice of comparators {6b}

We will conduct a two-phase trial to assess the effects of medical cannabis and alcohol on real-world driving performance. Phase 1 is a semi-naturalistic trial in which patients will complete driving assessments at two separate closed-circuit tracks (highway/urban driving environments) before and after consuming a standard dose of their prescribed cannabinoid medication. We decided not to institute a placebo control for patients as it was deemed unethical to withhold a patient’s treatment and because adequate blinding would be near impossible. In the absence of a placebo control for patients, we will implement an external control in the form of a secondary study (phase 2): a randomised, double-blind, placebo-controlled trial in which healthy volunteers will complete the exact same assessments at the same two tracks before and after consuming either alcohol (0.05% BAC) or placebo. Alcohol was chosen as the external control intervention for two reasons: (1) given the wide range of cannabinoid medications that we expect patients to be using, it would be impossible to select a ‘comparable’ dose or formulation for each participant and (2) because the dose to be administered is equivalent to the legal threshold for drivers in Australia (and numerous other countries) and thus has real-world relevance for road safety.

Phase 1 will involve 72 male and female patients (*n* = 36/*n* = 36) who are prescribed a THC-containing medical cannabis product (32 mg THC) to treat or manage chronic pain (*n* = 24), anxiety (*n* = 24), or insomnia (*n* = 24). These three conditions are the most common health indications for medical cannabis prescribing in Australia [1]. Patients will be further enrolled in a 1:1 ratio based on primary route of medical cannabis administration (inhaled or oral) and will complete assessments at different timepoints accordingly to account for differences in the pharmacokinetics and pharmacodynamics of inhaled and orally administered cannabinoid products [12]. This three-level stratification by gender, health condition, and route of administration will result in cell sizes of *n* = 6 (i.e. *n* = 6 males using an oral product for chronic pain), though this is for the purpose of maximising patient representation only and all patients will be treated as a single group for all primary statistical analyses. All patients will complete two testing days, including 1 day assessing highway driving ability and another day assessing urban driving skills. Treatments will be consumed by participants as prescribed by their physician in a supervised setting in the presence of a nurse.

Phase 2 (*healthy controls*) will involve 24 healthy male and female adults (*n* = 12/*n* = 12) who will complete a randomised, double-blind, placebo-controlled crossover trial to assess the effects of alcohol relative to placebo on real-world driving performance. Participants will complete two testing days per condition, 1 day assessing highway driving ability and another day assessing urban driving skills (i.e. four testing days total) during which they will receive alcohol (0.05% BAC) and placebo. Treatments will be administered orally (alcohol or placebo drink) using an established alcohol dosing formula in a supervised setting, with participants receiving treatments in a randomised and counterbalanced order.

## Methods: participants, interventions, and outcomes

### Study setting {9}

This trial will be conducted at Swinburne University of Technology and at two separate test sites: (1) METEC Driver Training facility and (2) Australian Automotive Research Centre (AARC). METEC is a driver training facility with 5 km of private closed roads and built-in traffic features including intersections, traffic lights, and roundabouts. AARC is an automotive proving ground with a 4.2-km-long two-lane highway circuit that follows the natural topography of the land and has a speed limit of 100 km/h. AARC is located in Wensleydale, approximately 2 h out of Melbourne. Over the course of the trial, and in both phases 1 and 2, participants will attend these two test sites for separate assessments of urban driving performance (METEC) and highway driving performance (AARC).

### Eligibility criteria {10}

#### Phase 1

For patients, inclusion criteria are as follows: (1) current medical cannabis prescription for chronic pain, anxiety, or insomnia persisting > 6 months, (2) on a stable dose of medical cannabis (≥ 3 months) with≥ 2 mg of THC/dose, (3) aged > 21 years, (4) in possession of a current and unrestricted Victorian driver licence, and (5) able to consume medical cannabis during the day as required for testing. Exclusion criteria are as follows: (1) On an unstable dose of medical cannabis or dose titrating, (2) unstable use of other medications that could impair driving, (3) unable to attend the test facilities for full-day testing sessions and stay overnight in a hotel for 2 nights, and (4) pregnant or lactating.

#### Phase 2

For healthy volunteers, inclusion criteria are as follows: (1) previous history of alcohol consumption in a single drinking session to an estimated BAC of 0.05% with no known adverse reaction [more than two standard drinks (female) or three standard drinks (male) on a single occasion], (2) under 100 kg (for weighted alcohol dose), (3) aged > 21 years and in possession of an unrestricted driver’s licence, and (4) oral fluid negative for all illicit drugs on the morning of test sessions. Exclusion criteria are as follows: (1) Use of cannabis > 1×/week; (2) AUDIT score (> 12) indicating problematic drinking; (3) use of any psychoactive medication that could impact driving; (4) current diagnosis of any Axis 1 mental health or substance use disorder; (5) taking any form of medication within 1 week of admission (except for routine medications to treat benign conditions); (6) unable to participate in scheduled visit, treatment plan, test, and other study procedures according to the protocol; (7) moderate-severe current depression (Beck Depression Inventory score of ≥ 20); (8) severe current anxiety (Beck Anxiety Inventory score of ≥ 16); (9) current participation in any other studies involving investigational or marketed products within 30 days prior to the screening visit; and (10) currently under administrative or legal supervision.

### Participant timeline {13}

#### Phase 1

**Fig. 1:**
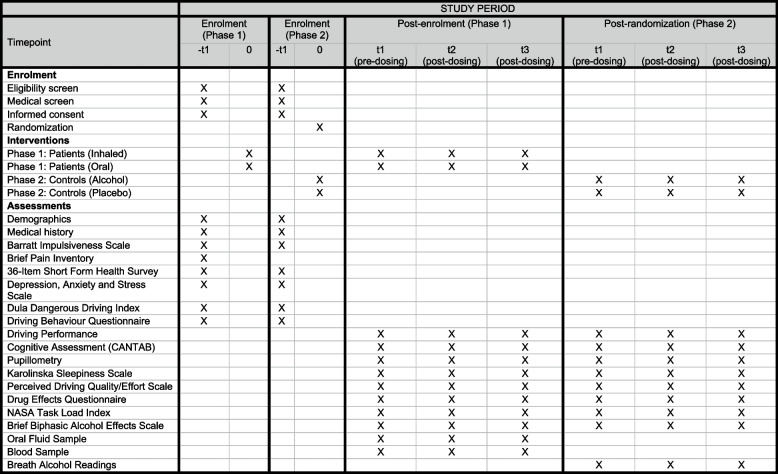
SPIRIT figure — schedule of enrolment, interventions, and assessments

In phase 1, patients will first complete a practice session and a baseline drive before completing two separate days of testing, including one test day at AARC and one test day at METEC. Due to the remote location of the AARC test track, participants will be transported by the research team and will stay in a hotel for 2 nights either side of the AARC test day. The test day schedule (see Fig. 1) will be identical for the two test days at AARC and METEC. Patients will complete three 20-min assessments of real-world driving performance over the course of each test day: one assessment (drive 1) prior to medical cannabis self-administration and two assessments (drives 2–3) after medical cannabis self-administration to assess acute effects. The timing of drives 2 and 3 will differ slightly depending on the route of administration to account for differences in cannabinoid pharmacokinetics and, by extension, the typical time course of expected behavioural effects (i.e. onset of effects, time to peak effects, and duration of effects).

For patients using oral products (*n* = 36), drive 1 will occur prior to medical cannabis self-administration, with drives 2 and 3 occurring 1.25 and 3 h after medical cannabis self-administration. For patients using inhaled products (*n* = 36), drive 1 will occur prior to medical cannabis self-administration, with drives 2 and 3 occurring 0.5 and 2.25 h after medical cannabis self-administration. Informed by prior work [13], drives 2 and 3 both fall within the potential window of impairment associated with inhaled and orally administered THC, with drive 2 corresponding to the expected period of peak effects, when impairment—if present—is likely to be greatest [14]. Blood and oral fluid samples will be collected immediately prior to each drive. An impairment assessment battery comprising cognitive testing and subjective drug effect questionnaires (see Outcomes {12}) will be administered immediately following each drive.

#### Phase 2

In phase 2, participants will first complete a practice session and a baseline drive before completing four separate days of testing, including two test days at AARC and two test days at METEC. Due to the remote location of the AARC test track, participants will be transported by the research team and will stay in a hotel for 2 nights before each of the two AARC test days before being transported back to Melbourne on the afternoon of the second AARC test day. Participants will then, in that same week, complete the two test days at METEC. Given the considerable logistical complexities associated with testing at remote locations, and to minimise potential dropouts, test days will be scheduled to occur back to back, meaning the washout period between test days will be minimal but within the expected metabolic elimination window for acute low doses of alcohol. As in phase 1, participants will complete three driving assessments within each test day, including a baseline drive in the morning (drive 1) followed by a second drive at the time of expected peak alcohol impairment (drive 2) and a third drive when impairment is expected to be subsiding (drive 3). The test day schedule (see Fig. 1) will be identical for each of these four testing days. The order in which participants receive alcohol and placebo at the two tracks will be randomised and counterbalanced.

### Sample size {14}

For phase 1, a sample size of *n* = 72 will allow us to detect a small magnitude change (*f* = 0.15) in our primary outcome measure—the standard deviation of lateral position—with 90% power assuming one group (i.e. all patients collapsed into a single group), six repeated measurements (three drives over 2 days), an estimated correlation among repeated measures of 0.6, and a non-sphericity correction of 0.6. The effect size used for this power calculation was derived from a previous on-road driving study with synthetic THC (dronabinol) [15] and conservatively reduced to account for the fact that treatment effects associated with prescribed medical cannabis may be considerably smaller than those seen previously in healthy volunteers given an intoxicating dose of THC. For phase 2*,* a sample size of *n* = 24 will allow us to detect a medium effect size (*f* = 0.25) with 90% power assuming one group, 12 repeated measurements (12 drives over 4 days of testing), an estimated correlation among repeated measures of 0.5, and a non-sphericity correction of 0.6. This effect size (*f* = 0.25) is equivalent to the level of impairment considered meaningful in a recent meta-analysis of 80 publications comprising 1534 driving or cognitive performance outcomes [13]. All sample size calculations were done in G*Power 3.1.

### Recruitment {15}

Participants will be recruited through dedicated practitioner channels, cannabis clinics in Melbourne, word o -mouth, and official recruitment streams (printed and online advertisements).

### Interventions {11} and allocation {16a, 16b, 16c, 17a, and 17b}

#### Phase 1

Patients will self-administer a standard dose of their prescribed cannabinoid medication at the time specified in the test day schedule (see Fig. 1). Self-administration will be witnessed by the study nurse who will confirm that the dose administered is consistent with instructions on the pharmacy label. In the case that patients report using more than one cannabinoid medication, they will be asked to use the medication that they would be most likely to use at that time of day, as patients often report using different types of cannabinoid products during the day and at night. Patients who would not normally take their cannabinoid medication during the day (e.g. patients with a sleep disorder) will be asked to use whatever their primary cannabinoid medication is, noting that the primary aim here is to assess the acute effects of that medication and that it would be impossible to organise test sessions around patients’ individual medication schedules.

#### Phase 2

Participants will be administered placebo or alcohol to achieve a target BAC of 0.05%, with an expected peak at 30–45-min post-ingestion. This will be achieved using an adjustable calculation based on the Widmark formula. Widmark’s formula states that one’s blood alcohol content is equal to the number of ounces of alcohol consumed, multiplied by a constant—for men by 3.75 and for women by 4.70—and then divided by that person’s weight. The formula is as follows: BAC = [alcohol consumed in grams/(body weight in grams × R)] × 100. For an average of 70-kg male person to achieve 0.05% BAC, this will require dosing of ~100 g of 40% alcohol or 3.7 standard drinks (30mL shots) with 201-g mixer orange juice (total drink 302 g). Placebo will be non-alcoholic vodka and orange juice, which we have effectively used to blind participants in previous studies. Non-alcoholic vodka is a clear, colourless liquid that is flavoured and formulated to mimic the taste and aroma of traditional vodka but contains no alcohol. Treatments will be prepared by the unblinded study nurse and given to participants in a stainless-steel mug with a lid and a straw to minimise potential unblinding during treatment administration. Allocation to treatment order (alcohol/placebo) will be randomised and counterbalanced according to a randomisation schedule held by the nurse and the study medical doctor. In the case of a serious adverse event occurring, emergency unblinding will be performed by the study nurse.

### Outcomes {12} and data collection methods {18a}

#### Demographics, participant characteristics, and medical history

At an initial screening session prior to testing, participants will undergo a comprehensive medical history review and screening questionnaire. The medical history review will collect information about age, sex, race/ethnicity, height, weight, body mass index, employment status, alcohol and other drug use, medical cannabis use history (for phase 1 only), past/current treatments, and concomitant medications. Participant will also complete the Barratt Impulsiveness Scale (BIS) [16], Brief Pain Inventory (BPI; phase 1 only) [17], 36-item Short Form Health Survey (SF-36) [18], Depression, Anxiety and Stress Scale (DASS-21) [19], Dula Dangerous Driving Index (DDDI) [20], and the Driving Behaviour Questionnaire (DBQ) [21].

#### Driving performance metrics

The primary outcome measure for both phases of the trial is the standard deviation of lateral position (SDLP), a measure of lane weaving, swerving, and overcorrecting that is highly sensitive to alcohol and drug effects and is widely considered to be the gold-standard measure of driving performance in human psychopharmacology research [22–24]. SDLP and all secondary driving performance metrics are described below in Table 1. The analysis metric is change from pre-dosing to post-dosing (i.e. t2–t1 and t3–t1) for all measures.

**Table 1:** Description of driving performance outcome measures

Domain	Measure	Outcome(s)
Driving performance (objective)	Standard deviation of lateral position (primary outcome)	Standard deviation of vehicle lateral position in centimetre (mean)
	Number of lane excursions	Number of times (count) one or more vehicle wheels exceeds the lane boundary
	Speed adherence	Mean speed (km/h) during driving, standard deviation of speed (km/h), and % time above the speed limit
	Steering variability	Steering reversal rate (number of directional changes per minute) and steering entropy (standard deviation of steering angle)
	Harsh acceleration	Count of rapid longitudinal acceleration events where acceleration is ≤ 0.35 g and < 0.58 g (harsh) or ≥ 0.58 g (very harsh)
	Harsh braking	Count of harsh braking events where deceleration is ≤ −0.75 g (extremely harsh), < −0.75g and ≤ −0.5g (very harsh) or ≤ −0.45g (harsh)
Ocular behaviour	Gaze behaviour (entropy)	Stationary gaze entropy and gaze transition entropy
	Rate and duration of visual fixations	Number of fixations (count per minute) and time of fixations (ms) where driver holds their gaze at a specific point on the roadway
	Microsleeps	Frequency (count) of long eye closures lasting ≥ 500 ms (NB: also adverse event indicator)
Driving performance (adverse event)	Instructor termination of drive	Number of times (count) a drive was terminated by the driving instructor
	Instructor intervention during drive	Number of times (count) the driving instructor had to intervene during a drive
Driving performance (subjective)	Instructor rating of fitness to drive	Instructor rating of fitness to drive (yes/no) at the end of each drive (proportion)

Driving-related outcome measures will be collected using two specially instrumented dual-control Mazda CX-5 research vehicles equipped with a proprietary automotive grade driver monitoring system and three GoPros to continuously and simultaneously monitor the forward roadway, the cabin interior, and the lane position of the vehicle. Drive terminations and assessor-rated driving performance will be scored by a qualified driving instructor who will always accompany participants while driving to ensure their safety.

#### Impairment assessment

The impairment assessment, completed after each drive on each test day (see Fig. 1), comprises assessments of cognitive performance, a pupillometry assessment, and measures of subjective state as outlined below.

##### Cambridge Neuropsychological Test Automated Battery (CANTAB)

Participants will complete three cognitive tests drawn from the CANTAB (Cambridge Neuropsychological Test Automated Battery, Cambridge Cognition, Ltd., UK) system designed specifically for neuropsychological testing and academic research. Tests include (1) Rapid visual information processing (RVP), an assessment of sustained attention; (2) reaction time (RTI), an assessment of motor and mental response speed and accuracy; and (3) spatial working memory (SWM), a visuospatial working memory task with notable executive function demands. Cognitive data will be analysed as change from t1 to t2 and from t1 to t3. All CANTAB assessments will be administered on an iPad in a quiet area of the testing site. Each task and its associated outcome measures are described below in Table 2:

**Table 2:** Description of CANTAB tests being used to assess cognitive function

Task	Measure	Outcomes
Rapid Visual Information Processing (RVP)	A sensitive measure of sustained attention (focus) which is integral to safe driving behaviour	A prime: A measure of signal detection sensitivity to the target sequence. Higher scores indicate better performance Response latency: The median response time (ms) for correctly identified target sequences Probability of false alarm: The proportion of incorrect responses, calculated as the number of false alarms divided by the total number of false alarms plus correct rejections
Reaction time (RTI)	Measures motor and mental response speed, including reaction time, and response accuracy. Relates to driver ability to respond quickly and appropriately to visual stimuli	Reaction time: Median time (ms) taken to release the response button after stimulus presentation. Calculated across correct trial Total errors: Total number of trials in which a response error occurred
Spatial working memory (SWM)	Measures visuospatial working memory, placing demands on executive function and assessing strategy use and errors. Reflects visual search efficiency in drivers as well as executive function which incorporates key cognitive skills	Between errors: Number of times a participant revisits a box where a token was previously found. Calculated across four, six, and eight token trials Strategy (6–8 boxes): Number of times a participant starts a new search from the same box they started with previously. Lower scores indicate greater strategy use

##### Pupillometry

Spontaneous and involuntary pupil movement will be evaluated using the F3D Fit-for-Duty test (AMTech Pupilknowlogy, Dossenheim, Germany). The F3D uses head-mounted binocular video goggles to assess fatigue and drug effects. The two pupillometry outcomes to be assessed include the pupillary light reflex (PLR), a measure of the pupillary response to light being used to assess potential drug impairment, and pupillary unrest index (PUI), a measure of pupillary oscillation being used to assess fatigue. The PUI and PLR values are generated automatically by a proprietary algorithm and will be analysed as a proportion of participants showing either impairment or fatigue per timepoint.

##### Subjective assessments and questionnaires

Participants will complete a suite of subjective state measures during each impairment assessment. These include the Karolinska Sleepiness Scale (KSS) [25], Perceived Driving Quality/Effort Scale (PDQ/PDE), Drug Effects Questionnaire (DEQ), and NASA Task Load Index (TLX) [26]. In phase 2, participants will also complete the Brief Biphasic Alcohol Effects Scale (B-BAES) [27]. Mean scores on the KSS, PDQ/PDE, DEQ, TLX, and B-BAES will be analysed as change from t1 to t2 and from t1 to t3. The TLX is not intended as a stand-alone measurement but rather an indicator of workload during the driving task, so results will be interpreted in relation to driving performance metrics as described above.

#### Oral fluid and blood

On each test day in phase 1, patients will provide three oral fluid samples and three blood samples. Oral fluid samples will be collected using the Securetec DrugWipe Twin. Blood samples will be frozen at −80 °C for long-term storage at Swinburne University and analysed later via liquid chromatography—mass spectrometry (LC–MS) for THC, 11-hydroxy-THC (11-OH-THC), 11-nor-9-carboxy-THC (11-COOH-THC), and cannabidiol (CBD) by the Victorian Institute for Forensic Medicine (VIFM) according to previously published methods [28]. In phase 2, participants will provide breathalyser samples throughout the day (as shown in Fig. 1) to obtain BAC readings. Cannabinoids’ concentrations and BAC readings will be analysed as mean (SD) or median (IQR) values at each timepoint.

### Data collection {18b} and management {19}

Our staff are highly trained in the collection, management, storage, and confidentiality of data to ensure compliance with all policies and regulations. Identifiable patient data will be stored electronically in password-protected files only accessible to study investigators. Coded paper records will be kept in designated, locked storage areas. Study case report forms are primarily electronic and hosted on Qualtrics and will be regularly reviewed for completeness and compliance. Driving data will be checked for completeness of recordings and backed up after each session. All other data will be backed up at regular intervals to minimise the risk of data loss.

### Statistical analysis {20a, 20b, 20c}

Participant demographic and baseline characteristics will be summarised separately by study phase and treatment condition, where applicable. Continuous variables will be summarised using means and standard deviations (SD), and where appropriate, medians and interquartile ranges (IQR) will also be reported. Categorical variables will be summarised using frequencies and percentages (%). No formal hypothesis testing of demographic variables will be conducted; summaries are intended to describe the study population and assess baseline comparability across groups. Analysis of primary and secondary outcome measures will be conducted using a series of linear mixed-effects models (continuous data), Poisson or negative binomial generalised linear mixed models (count data), logistic mixed-effects models (binary data), or time-binned mixed models and linear regression (ocular data). Post hoc tests with Bonferroni adjustments will be conducted where significant main effects or interaction effects are observed. No deletion or imputation will be used where data is missing at random, as mixed models inherently accommodate incomplete repeated measures. Sensitivity analysis will be conducted using delta-adjustment for nonrandom missing data. Exploratory subgroup analyses will test for differences in change in outcome measures over time as a function of health condition, route of administration, and gender (phase 1 only). All statistical analyses will be conducted using SPSS (IBM Corp, Armonk, NY, USA) and R, and tests will be set two-tailed with a conventional level of significance of *p* < 0.05.

### Data monitoring {21a, 21b}

Given the low-risk nature of the interventions in this study, a formal independent Data Monitoring Committee was not established. Instead, trial oversight, including safety and data integrity, is managed internally by the study team and through fortnightly meetings with the trial sponsor.

### Harms {22}

Any serious adverse events or protocol deviations will be reported to the Ethics Committee in accordance with regulatory requirements.

### Auditing {23}

No formal trial audits are planned, though study investigators will meet fortnightly with the study sponsor to review trial progress, recruitment, and any operational difficulties. An internal steering committee at the university will also meet on an ad hoc basis to provide project management oversight and review budgets, timelines, and alignment with contractual obligations.

### Ancillary and posttrial care {30}

No posttrial care is planned unless adverse events necessitate further observation. In the event of trial-related serious injury, participants may be eligible for compensation in accordance with relevant laws and policies.

## Discussion

Our primary objective in this two-phase trial is to evaluate the effects of prescribed medical cannabis on real-world driving performance and to provide an ecologically valid point of reference for these results by assessing the effects of alcohol on real-world driving performance at a threshold legal BAC limit. The complexities of prescribed medical cannabis use necessitate a unique trial design that considers different health indications that patients may be prescribed medical cannabis for and the wide range of medical cannabis products that patients may be using to manage their symptoms.

This is the first trial to assess the impact of prescribed medical cannabis on real-world driving performance. There is no current standard for assessing ‘driving performance’ in a real-world environment, and ‘driving performance’ is a broad term that captures the many cognitive faculties and motor skills involved in safely handling a vehicle. Our team has developed a unique set of hardware and software instruments to capture subtle changes in driving performance that have been through a rigorous process of validation and field testing. The results of this validation work will be presented alongside trial findings when these results are published and disseminated.

This trial will determine whether people prescribed medical cannabis exhibit impairment or changes to their driving performance after consuming a standard dose of their prescribed medication. The on-road driving data outcomes will be supported by a suite of related assessments (e.g. cognitive performance and biological matrices) that will be used to comprehensively define levels of impairment and identify useful indicators of impaired performance and/or altered driver state (e.g. altered ocular activity). These data will help to inform health and road safety policy.

## Trial status

Participant recruitment began in October 2024 and is expected to be complete in mid-2026.

## Supplementary Information

Additional file 1: SPIRIT checklist.

Additional file 2.

## Data Availability

Trial results will be communicated to participants and disseminated to the broader community through publication. Authorship will be determined based on intellectual input and contributions to data collection, data analysis, or write-up of results. Researchers can contact the principal investigator for requests to access the full study protocol or participant-level data or statistical code.

## Appendix

### Informed consent materials {32}

The informed consent forms are attached as supplementary material.

## Abbreviations

11-COOH-THC: 11-Nor-9-carboxy-THC
11-OH-THC: 11-Hydroxy-THC
AARC: Australian Automotive Research Centre
ANZCTR: Australian New Zealand Clinical Trials Registry
AUDIT: Alcohol Use Disorders Identification Test
B-BAES: Brief Biphasic Alcohol Effects Scale
BAC: Blood alcohol concentration
BIS: Barratt Impulsiveness Scale
BPI: Brief Pain Inventory
CANTAB: Cambridge Neuropsychological Test Automated Battery
CBD: Cannabidiol
DASS-21: Depression, Anxiety and Stress Scale
DBQ: Driving Behaviour Questionnaire
DDDI: Dula Dangerous Driving Index
DEQ: Drug Effects Questionnaire
KSS: Karolinska Sleepiness Scale
PDQ/PDE: Perceived Driving Quality/Effort Scale
PLR: Pupillary light reflex
PUI: Pupillary unrest index
SDLP: Standard deviation of lateral position
SF-36: 36-Item Short Form Health Survey
THC: Delta-9-tetrahydrocannabinol
TLX: NASA Task Load Index
VIFM: Victorian Institute for Forensic Medicine
