# Supplementary material for: Effects of prescribed medical cannabis and alcohol on real-world driving performance (CAN-TRACK): a study protocol for a two-phase trial
Source: Trials. 2026 Feb 6;27:200. doi: 10.1186/s13063-026-09512-x (PMC12977860; doi:10.1186/s13063-026-09512-x)
Supplement: Supplementary file 2 — Additional file 2. [file 13063_2026_9512_MOESM2_ESM.pdf]

SWINBURNE UNIVERSITY OF TECHNOLOGY
Centre for Mental Health and Brain Sciences (CMHBS)
Participant Information and Consent Form (Arm 1)

Full Project Title: A Closed-Circuit Track Trial to Assess Risk, Impairment and Performance from Medical Cannabis (CANTRACK)

Protocol Number: V1.6

Study Arm: 1 (Patients)

Principal Researcher(s): Prof. Luke Downey, Dr Thomas Arkell, A.Prof Amie Hayley

Student Researcher(s): Ms Tayla Amiguet

Research Staff: Dr Brooke Manning, Dr Xinyun Hu, Mr Simon Hayes, Dr Blair Aitken

Research Nurse: Ms Maggie Bell, Mr Eugene Jandoc

Medical Officer: Prof. Edward Ogden

Trials Coordinator: Ms Rebecca King

Project Manager: Pouria Amiri

1. Your Consent

You are invited to take part in this study which investigates how prescribed doses of medical cannabis impact on-track driving performance. This Participant Information and Consent Form (PICF) will explain what is involved in the study, and what your rights and responsibilities are as a participant. Knowing what is involved will help you decide if you want to take part in the study. Please read this information carefully. Ask questions about anything that you don't understand or want to know more about. Before deciding whether to take part, you might want to talk about the study with a relative, friend or your local doctor. Participation in this research is voluntary; you do not have to take part if you do not want to, and if you do choose to take part you will be free to withdraw at any time. If you decide you want to take part in the study, you will be asked to sign the consent form. By signing it you are telling us that you:

- Understand what you have read.
- Consent to take part in the study.
- Consent to undergo the tests that are described.
- Consent to the use of your personal and health information as described.
- Understand that you will not be exempt from roadside drug testing laws once you leave the testing site.

You will be given a copy of this **Participant Information and Consent Form** once signed to keep.

2. What is the purpose of this research project?

Medical cannabis is increasingly used for its potential therapeutic benefits. There are a range of medical cannabis products that are available, including oils, extracts, and capsules, and these typically contain varying concentrations of delta-9-tetrahydrocannabinol (THC) and cannabidiol (CBD). Although medical cannabis can be accessed with a prescription, roadside drug testing laws in Victoria make it an offence to operate a vehicle with any detectable amount of THC in your system. This means that patients who are using THC-containing medical cannabis products are prohibited from driving.

While work has been undertaken to understand the impacts of THC on impairment, significant gaps exist in our understanding of the impacts of medicinal cannabis (containing THC) on driving performance. The Victorian Government has therefore committed to conducting a study to understand whether THC-containing medical cannabis impacts driving performance in a fully controlled environment. The outcomes of this study will add to the data and knowledge needed to determine if Victorians who are prescribed medicinal cannabis (containing THC) can be in control of a vehicle without compromising their safety, or the safety of other road users.

3. Am I eligible to participate in the study?

Below is a list of criteria that you will need to meet to be eligible to participate in the study.

To participate in the study, you must meet the following criteria:

1. In possession of a valid and current medical cannabis prescription for a health condition that has persisted for >6 months, including (1) chronic pain, (2) a mental health condition (anxiety), or a (3) sleep disorder
2. Have been using medical cannabis for 3 months or longer, and on a stable dose (meaning you are using the same amount each time on a regular schedule)
3. Using a medical cannabis product that contains at least 2 mg THC *per dose*
4. Aged >21 years of age
5. In possession of a current and unrestricted Victorian driver licence (i.e. no P's)
6. Able to consume medical cannabis during the day as required for testing.
7. Willing to abstain from the following prior to their scheduled visit:
    a. Do not take your prescribed medicinal cannabis treatment the morning *before* attending your testing session.
    b. No driving or riding a bicycle or motorbike from the testing site
    c. No driving, riding or operating heavy machinery for 12 hours after leaving the site

Participants presenting with any of the following **will not** be included in the trial:

1. Use of prescribed medical cannabis for less than 3 months

2. Dose titrating (meaning you are still increasing the amount of medical cannabis you are using to find the optimal therapeutic dose)
3. Unstable use (i.e., infrequent use or dose titrating) of other medications that could impair driving such as opioids or benzodiazepines
4. Unable to attend the test facility for a whole day and stay overnight for 2 nights (relevant for one of the driving sites only)
5. Pregnant or lactating.

A medical doctor will review your medical history in detail to decide whether you meet the eligibility criteria for inclusion for this trial. If you are deemed ineligible due to any of the above criteria, you will have the opportunity to speak with the doctor so that they can provide a more detailed explanation about why you will not be able to take part. You will also have the opportunity to be referred to the Swinburne Psychology Clinic and/or Lifeline.

4. What does participation in this research involve?

PLEASE READ THIS SECTION CAREFULLY TO ENSURE YOU FULLY UNDERSTAND WHAT YOUR PARTICIPATION WOULD INVOLVE.

Participation in the study will take a minimum of two weeks to complete and involves –

- A screening visit at Swinburne University at least one week prior to the study.
- A consecutive three-day / two-night stay for study testing (Monday to Wednesday).
- Another testing session (8:00 am to 4:30 pm) during the same week (Thursday / Friday).

The screening will take place at:

Swinburne University of Technology
Advanced Technologies Centre, Level 3
427 Burwood Rd, Hawthorn,
VIC, 3122

The testing will take place at two driver-training facilities:

1. **Australian Automotive Research Centre (AARC)** (https://aarconline.com/)
   445 Gum Flats Rd, Wensleydale, VIC 3241

2. **METEC Driver Training facility** (https://www.metec.com.au):
   112 Colchester Rd, Bayswater North, VIC, 3153

Participation in this study will involve first attending Swinburne University's Centre for Mental Health & Brain Sciences for a medical screening. This screening visit will take place at least one week prior to travelling to both AARC and the METEC Driver Training facility for testing. If you

meet eligibility criteria and decide to participate, you will need to be available for four days within a single week (Monday, Tuesday, Wednesday, and Friday). As AARC is located several hours from Melbourne, you will be required to arrive at Swinburne University on Monday afternoon to be transported to the testing facility and **stay overnight for 2 nights** at accommodation that will be provided to you at no expense. Transport will be provided back to Swinburne University on Wednesday morning. On Friday, you will be required to attend Swinburne University and will be provided with transportation to and from the METEC Driver Training facility for testing. Before we enrol you in the study, we will ask you to complete an online pre-screening questionnaire. This will help us decide if you might be eligible to take part and will minimise the time you need to spend on-site at Swinburne University of Technology. This online questionnaire can be completed remotely at home and will take approximately 20 minutes to finish. The following section describes each of the events in detail.

4.1 Online Screening Questionnaire

Location: *Remote (online)*

Before we can enrol you into the study, we will conduct an initial pre-screening assessment to make sure you will be eligible to take part. We ask that you complete an online questionnaire, which will take about 20 minutes. We will first ask whether you have read this PICF document (you are required to do so before completing the pre-screening questionnaire). Following this, we will ask you to confirm your consent to proceed with the survey. If you do not want to proceed to the question portion of the survey, you may exit the webpage by clicking ‘no’ (or closing your internet browser), and no further information will be collected. If you agree to continue with the survey, we will then ask you some further questions to determine whether you meet the study’s eligibility criteria.

After completing this pre-screening questionnaire, you will be asked to consent to being contacted by the research team. If you do not want to proceed or be contacted by the research team, you may exit the webpage by clicking ‘no’ (or closing your internet browser), and no further information will be collected. If you agree to continue, we will request a contact number and email from you, and one of our researchers will call you for a brief phone call to reconfirm the details you provided and make sure that you are happy to proceed with the in-person screening visit (see below). During this call, if you are happy to proceed, we will schedule the in-person consent and medical screening visit. This call will take approximately 10 minutes. We will send you a reminder email with the screening visit date and time, and what you will need to bring on the day.

4.2 Screening Visit (V0)

Location: *Level 3**,** Advanced Technologies Centre (ATC), Swinburne University of Technology, Hawthorn.*

Before we can enrol you into the study, you will first be required to give voluntary written and informed consent. We will again explain the study aims, requirements and potential risks, and you will be asked to sign the Consent Form (at the end of this document). Two copies of the Consent Form will be signed, one for the study records and one for you to keep.

You will then be required to attend a medical screening with the research nurse. During this screening, the research nurse will ask you about your medical history in detail and review your past use of cannabis, alcohol, and other drugs. To assist, you should bring with you any health records you have on file that may be relevant (e.g., notes from doctor appointments, hospital records). You will be required to discuss the details of your medical cannabis history (e.g., how long you have been using it for, what sorts of products you have used) and demonstrate that you have held a valid prescription for at least 3 months. You will therefore need to show evidence of your prescription in the form of a physical or e-script, or medical cannabis product with a pharmacy label. The doctor may cross-reference this information against SafeScript, a Victorian database for monitoring prescribing of certain prescription medicines.

You will also be required to provide details about your current use of medical cannabis, including the types of products you use, how frequently you use them, how you self-administer them (i.e., dropper or vaporiser), and how much you use at a time. All information collected during this screening will be kept entirely confidential and will only be accessible to the study nurse, doctor and principal investigators. This screening visit will take approximately 30 minutes to complete.

If the research nurse decides that you are **not** eligible, there is nothing further that you will need to do.

If the research nurse decides that you **are** conditionally eligible, you will be required to remain at the site for approximately 20 minutes to complete several demographic related questionnaires that will be confirmed by our study physician. We will also tentatively schedule your upcoming study visits which will occur within a single week from Monday to Friday. We understand that this time window is relatively short and that you may be required to take time off work in order to participate in this study. We can provide a note stating that you will be taking part in a research trial, and you will be compensated for your time (see Section 16). Following this visit, the study doctor will review the medical history collected by the research nurse and confirm whether you are eligible or not to participate in the active testing part of the study. We will contact you after your screening visit to confirm your testing visits.

4.3 Testing Visits (V1 – V2)

There are two testing days (Tuesday and Friday) that will each run for approximately 6-8 hours depending on whether you have been prescribed an oral (e.g., oil) or inhaled (e.g., vaporised) medical cannabis product. Study testing visits will commence at approximately 8:00 am and finish

no later than 4.30 pm. On these testing days, you will be completing various assessments before and after consuming your prescribed medical cannabis. We recognise that these are long days, so we will provide food and beverages throughout the day (including lunch and snacks), and a space for you to relax during periods of downtime. On any given test day, you may be onsite with one other participant taking part in the same study. Because these testing visits are logistically complex and require considerable resources, the schedule has been designed in such a way as to allow testing of two participants on the same day. In addition to these two testing days, there will be two travel days (Monday and Wednesday). You will therefore need to be able to commit to **four days of travel/testing within a 1-week period** to take part in this study. The figure below outlines the schedule for the study:

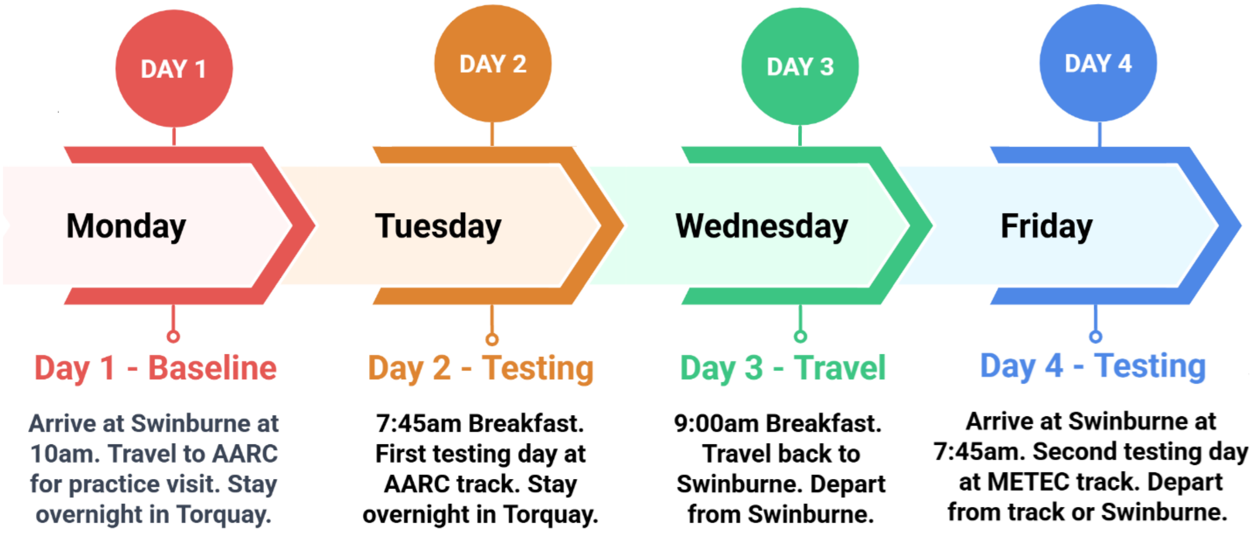

You will need to make sure you do not take any illicit drugs or medications (other than those you have been prescribed) for the one week-prior to, and for the entire duration of the study. You will not be allowed to consume alcohol for 24 hours before each study day (Monday/Tuesday and Friday). On the morning of each testing day, you will be breathalysed to confirm you have no alcohol in your system and you will be required to provide a saliva sample to confirm no recent use of drugs other than those prescribed by your doctor.

AARC (Australian Automotive Research Centre), Wensleydale

On **Day 1 (Monday)**, you will be required to meet study researchers at Swinburne University of Technology in Hawthorn at approximately 10:00 am on level 3 of the ATC building (where the screening visit took place). From there, you will complete a few questionnaires prior to being transported to AARC (approximately 2 hours away), with lunch provided along the way. At AARC, you will complete a training session to familiarise you with the vehicle, and a baseline driving and cognitive test. Later that afternoon, you will be transported to a dedicated research accommodation, approximately 35 minutes' drive from AARC. You will stay overnight in a private

room and will be provided with a choice of dinner options prior to the study being conducted the following day. On the morning of the test day **(Day 2, Tuesday)**, you will be provided with breakfast and then be transported to the AARC site at approximately 8:00 am. Testing will run for approximately 6-8 hours. At the end of the day, you will be transported back to your accommodation for an additional night's stay and will be provided with a choice of dinner options.

This means you will be required to stay at the accommodation in Torquay for 2 nights in total. The following morning **(Day 3, Wednesday)** at approximately 8 am, you will again be provided with breakfast prior to being transported back to Swinburne University of Technology in Hawthorn, from where you will be able to depart via taxi, uber, or lift.

When staying at Torquay, your accommodation will be booked for you at the following location:

Location: *Wyndham Resort Torquay, 100 The Esplanade, Torquay, VIC, 3228*

METEC Driver Training Course, Bayswater North

For the testing session at METEC **(Day 4, Friday)**, a member of the research team will arrange to meet you at Swinburne University of Technology in Hawthorn, where they will transport you to the METEC facilities in Bayswater North (approximately 40 minutes drive from Swinburne). You will have the option to depart directly from the testing location in Bayswater North via a taxi, uber, or lift- or opt to be accompanied back to Swinburne University in Hawthorn, from where you can secure a taxi, uber, or lift home.

During the baseline session (Monday) and the two testing days (Tuesday at AARC and Friday at METEC), you will be required to complete a range of tasks both before and after consuming a standard dose of your prescribed medical cannabis product (at approximately 11:00 am). These tasks are outlined below:

1. Driving Performance

At both AARC and METEC, you will complete three driving assessments each testing day running for approximately 20 to 25 minutes each. An additional practice drive to familiarise yourself with the vehicle will be completed at AARC on the Monday afternoon prior to arriving at the accommodation in Torquay. The AARC driving circuit is a 4.2 km highway track that follows the natural topography of the land and is typical of an Australian highway driving environment. It too is closed to ordinary traffic. The METEC driving circuit is approximately 5 km long, closed to ordinary traffic, and comprises both gentle and sharp curved closely resembling public roads typical of urban Australia. All driving tasks will be conducted in an instrumented car with automatic transmission and fitted with a double set of pedals (dual controls). A licensed driving instructor will be present in the front passenger seat during all driving sessions to intervene if necessary. We will be collecting a broad suite of measures while

you are driving allowing us to comprehensively measure driving performance in an on-road environment.

During the driving task, you will have a dash-mounted eye tracking device to capture your head and eye movements. The device will use an infra-red video to capture a video record of your face, head and eye movements and posture to determine where you are looking; whether you have your eyes open or closed, and what you are doing (e.g., hands on or off steering wheel, attending to driving or non-driving related tasks etc). This device will not interfere with the task, and you will not need to interact with the devices at all.

2. Cognitive Performance

You will be asked to complete several cognitive tests three times per test visit. These tests are designed to assess skills relevant for driving such as multitasking, visual processing, processing speed and reaction time. These will be administered on an iPad in a quiet room and will take approximately 25 minutes each to complete.

3. Questionnaires

Throughout each visit, you will be asked to complete a series of brief questionnaire that ask you to rate aspects of your current physical and mental state (e.g., how sedated do you feel right now?). Before and after the driving test, you will also rate your subjective alertness and perceived quality of driving performance.

4. Biological Sampling

The analysis of biological samples is necessary for this trial, and refusal to provide them is sufficient for exclusion from participating in the study. All biological samples will be taken by a registered nurse or qualified venepuncture technician.

a. Urine sample (female participants only): A screening urine sample will be obtained for us to assess the pregnancy status of all female participants. This will be conducted at baseline on Monday, and prior to each of the two testing sessions (Tuesday and Friday), under supervision of the research nurse. A new test will be used each time. If the test is positive (pregnancy is confirmed), you will not be eligible to complete the study, and will be counselled by the research nurse.
b. Saliva samples: Throughout the study, we will collect saliva samples using the same devices that are used for roadside drug testing. We will collect one sample on arrival to Swinburne on Monday, and three samples for each full testing day (Tuesday and Friday). This will allow to see whether you would test positive to a roadside drug test after consuming your medical cannabis. A new test will be used each time and may be repeated if the test result is unclear.
c. Blood samples: Throughout the study, we will collect blood samples to allow us to see how much THC is in your system before and after consuming your medical cannabis.

A small amount of blood (up to 10 mL) will be collected three times for each full testing day (Tuesday and Friday) by a registered nurse or venepuncture technician and will be frozen for analysis at a later date.

4.4 Exemption from Roadside Drug Testing Laws

In Victoria, driving with any amount of THC in your system is an offence. To allow this trial to take place, a special exemption has been put in place by the Victorian Government. This exemption means you can legally drive with THC in your system during this trial. This exemption **only** applies while you are driving at METEC or AARC under the active supervisions of both the research team and a licensed driving instructor. The exemption **does not apply** once you leave the testing site. If you were to operate a motor vehicle after leaving the testing site, you could be subject to penalties including a driver licence suspension and monetary fines. More information about driving while taking medical cannabis can be found on the VicRoads website: https://www.vicroads.vic.gov.au/safety-and-road-rules/driver-safety/drugs-and-alcohol/medicinal-cannabis-and-driving

5. What other options do I have?

You do not have to take part in this research study. Participation is voluntary and you are free to withdraw at any time. There is no penalty for withdrawal. Any data collected prior to withdrawal may be retained for analysis.

6. Who is conducting and paying for this research?

This study is funded by the Victorian Government and run by researchers at the Centre for Mental Health and Brain Sciences at Swinburne University of Technology.

7. What if something new comes up during the trial?

We will tell you about it. The study team or doctor will discuss with you what it means and ask whether you want to continue in the study. If you decide to continue in the study, we will ask you to sign an updated consent form.

8. Can the researchers stop the trial early?

Yes, it happens sometimes. If it does, the study doctor will let you know and explain the reason behind the decision.

9. What will happen to information about me?

The questions we ask you prior to your involvement in the study are to ensure there are no factors that could put you at risk during the study and to ensure you meet eligibility criteria. We will ask questions about your demographics, medical history, and previous drug and alcohol use. This information will be collected in a private room, and any identifiable documents will be stored securely in a locked filing cabinet at Swinburne University of Technology. You have the right to request that any incorrect information collected is corrected. Please note that confidential information collected as study data may be subject to inspection/review by authorised representatives from the Sponsor, regulatory authorities, Swinburne University Human Research Ethics (SUHREC), or as required by law.

Apart from this requirement, the information you provide us will not be disclosed to anyone else, unless the information you disclose puts you or another person at risk. If you ask, you will be provided with a copy of your height and weight results, so that you can add this information to your medical history or so that we can pass this along to your treating doctor (if you request and consent to this). Your name will not appear on any physical documents except the consent form. On all forms you fill out and all the data recording sheets; a ***de-identified*** number will be used. There will be one document with the names and matching numbers, which will be password protected and only accessible by the investigators.

The video that is collected during driving will be ***potentially identifiable*****,** as it will capture head pose, gaze and pupil metrics and eyelid opening in real-time. This information will be collected and held on-site at Swinburne University of Technology, before being provided to the study collaborator, Seeing Machines. This ***potentially identifiable*** information/data [video of record the driver's face, head and eye movements and posture to determine: where the driver is looking; whether the driver has his or her eyes open or closed and what the driver is doing (e.g., hands on or off steering wheel, attending to driving or non-driving related tasks etc.)] is collected and saved in a format that can be read by computer software accessible only by Seeing Machines. This means that the data cannot be accessed or read by any other computer programs, even those at Swinburne University. The data will be collected and sent to Seeing Machines via an existing secure and direct data transfer pipeline. The data file will be labelled with your unique identifier only. That means that there will be no identifiable information, such as your name, date of birth or any other information attached to the data file. No raw data, video or identifiable data will be made publicly available or shared with any other person or organisation not listed in this application. Summary data only will form the basis of journal article submissions and conference papers and reports that will be made publicly available at the conclusion of the study.

Aggregate health, medical and demographic data will be used by Ms Tayla Amiguet in fulfilment of the requirements of her Doctor of Philosophy (PhD) degree at Swinburne University of Technology, under the supervision of Prof. Luke Downey, A/Prof Amie Hayley and Dr Thomas

Arkell. No personal identifying information will be shared in this document and only de-identified information will be reported.

All biological samples (including saliva test results and blood samples), we will use a ***de-identified*** number to identify you. Blood samples will be labelled with your participant number as well as date and time of collection.

At the conclusion of the study, the names and matching participant numbers will be kept in separate locked filing cabinets and password-protected computer files stored on the secure university server. The data collected during the study will remain locked securely at the Centre for Mental Health & Brain Sciences for a period of at least 15 years. The data must remain accessible if an article is published. However, if after this time the data is no longer needed and nothing in relation to the study is being published, the data will be destroyed.

10. What will happen to my test samples?

Once the saliva screening test result has been noted, this test will be discarded. Blood samples will be destroyed immediately after analysis. The results of these tests may be used for future studies; however, your identity will not be disclosed.

11. What are my responsibilities during the trial?

If you agree to participate in this study, you agree to be responsible for following all instructions given by the researchers. You also agree to comply with the other conditions in this document. If you cannot, or do not wish to accept this responsibility, then we cannot accept you as a participant in the study. On each of the testing days we ask that you abide to the following test day restrictions:

- No alcohol within 24 hours prior to testing
- No illicit drug or medication use for at least 1 week prior to testing and for the duration of the trial
- No driving or riding a bicycle or motorbike to or from the testing site

For the testing day at AARC, you will be required to choose what you would like for dinner out of several possible options. Breakfast options will also be provided for you. Lunch and snacks will be provided at both METEC and AARC. While we will do our best to accommodate any dietary requirements you may have, we cannot promise that we will be able to accommodate everything. Please also note that while you will be permitted to use you medication as prescribed (e.g. an inhaled medical cannabis product is a medicine) on site at AARC, smoking (combustible or vaporised) cigarettes is not permitted. Smoking is also not permitted within the grounds of the accommodation. You are welcome to use non-smoking nicotine alternatives such as patches if

needed. Please refer to Section 22 for additional information about the responsibilities you have when it comes to sharing information about the study.

We are committed to working with our participants and partners to improve the experience and outcomes for everyone involved in our study. We work together as partners, treating one another with respect and trust. Participants have the right to feel emotionally and physically safe, to be listened to and treated with respect and dignity. Our staff also have the right to feel emotionally and physically safe. Participants and staff both have a responsibility to ensure their actions do not threaten or harm others.

If your behaviour or conduct endangers the wellbeing of the staff, our partners, or other research participants, your involvement in the research project will be terminated

12. What are the possible benefits?

There are no direct personal benefits to you from participating. You will receive a small monetary reimbursement to acknowledge your time.

13. What are the possible risks?

The screening process we ask you to go through prior to your involvement in the study is to ensure there are no factors that could put you at risk during the study. Part of this screening process requires some personal information about medical issues and possible criminal and/or illegal behaviour or activities such as any illegal drug use and drug addiction. If you are female, you will also be required to undergo a pregnancy screening test, which may indicate that you are pregnant.

Medical treatments, including medical cannabis, can cause side effects. As you will be self-administering a medication that you already take on a regular basis, we do not anticipate any additional risks associated with taking your medication during this trial. Nonetheless, as part of this trial, you may be required to take your medication at a different time of day to when you might usually take it. You could experience new or unusual symptoms as a result of this. You should tell the study nurse who will be onsite immediately if you experience any new or unusual symptoms.

If you decide to leave either of the two testing sites before the testing procedures have concluded, you will be required to sign an indemnity form. This will be used to show that you understand the potential risks involved in leaving the testing site prematurely, despite the advice of the nurse and research staff. You will keep a signed copy, and the researcher will keep a signed copy.

14. Can I have any other procedures during this trial?

You can continue taking any medications that have been prescribed to you by a doctor. You must tell us about any procedures or medicines you may begin taking in the lead up to or during the

study. This is in your interest as well as being important for the study. You must tell us about any over-the-counter medications, vitamins, or herbal remedies you are taking and about acupuncture or other alternative procedures because they may interact or interfere with study procedures. You must also tell us about any changes to these while you are participating in the study.

15. What if I withdraw from this research project?

If you decide to withdraw, please notify a member of the research team. Your participation in this study is voluntary and you are free to withdraw from the study at any time. If you arrive on time for your testing session, and have adhered to the study restrictions, but then withdraw from the study, you will still be reimbursed for each testing session you have completed. If you decide to withdraw during the test day at AARC, you will be required to remain onsite until the end of the day due to its remote location. Any data collected before your withdrawal may still be included in the project.

16. Will you pay me to participate in the research project?

Upon completion of the study, you will be compensated for your time commitment by electronic funds transfer to a nominated bank account, up to a total of $1400. This includes $500 per test day (Tuesday and Friday), and $200 for the two travel days (Monday/Wednesday). In addition to this, the cost of travel home from each testing session will be available using Eftpos or taxi vouchers. These taxi vouchers ($50 per session, for a total of $100) will be provided upon request at the end of each testing session. If vouchers for travel home are not required, this sum will be added to your reimbursement for a total of up to $1500. Travel to the testing facility or to Swinburne University for each session will not be reimbursed. If you withdraw before you finish the study, you will be reimbursed for each test day that you have successfully completed while eligible. Payments will be handled by Swinburne University of Technology who process bank transfers once monthly on the last week of each calendar month. Please be advised that depending on when you complete your testing week, it may be from two to six weeks prior to receiving funds to your nominated bank account.

17. What happens when the trial ends?

A summary of the overall group findings will be made available to you via email or mail upon completion of the study and data analysis. This summary will not include individual results, only aggregate group data.

18. Will my data be used for purposes beyond the extent of this study?

There is the possibility that the data collected in this study may be combined with datasets from similar studies or used for future, related projects. For example, to compare blood THC concentrations with levels observed in other studies. The information collected in this project will

only be shared for research purposes. In any future research that will use this data, your identity will not be disclosed, and you will not be contacted by the researchers of the future project. In the case that a short video segment was to be made available to media or promotional videos using the video data collected here, explicit and separate written and informed consent will be obtained by the research team.

19. How can I access my information?

In accordance with relevant Australian and/or Victorian privacy and other relevant laws, you have the right to access the information collected and stored by the researchers about you. You also have the right to request that any information, with which you disagree, be corrected. Please contact one of the principal investigators if you would like to access your information.

20. Your safety

Your safety during the testing sessions is our main priority. There will be a registered nurse onsite throughout the testing session, and a study doctor (Prof Edward Ogden) will be on call at all times, in the case of a medical emergency. During the on-track driving tasks, you will be always accompanied by a licensed driving instructor. The instructor will have access to dual controls allowing them to take control of the vehicle if necessary. Additional strict safety protocols will be in place at all times to ensure your safety at both test tracks and whilst in transit.

21. Is this research project approved?

The ethical aspects of this research study have been reviewed and approved by Swinburne University Human Research Ethics (SUHREC). This study will be conducted according to the National Statement on Ethical Conduct in Human Research (2023) produced by the National Health and Medical Research Council of Australia (NHMRC). This document has been developed to protect the people who agree to participate in human research studies.

22. Information shared by participants

If you participate in this study, you acknowledge and agree that:

- Any views or opinions expressed by you associated with your participation in this study (if any) are strictly your own. If applicable, you must ensure that you make it clear that you are expressing your own views and that they should not be perceived as an official comment about the study.
- The Victorian Government does not endorse any views or opinions expressed by you about the study and they do not represent the Victorian Government's position on the subject matter.

- You will not take any photos or videos at AARC. This is a condition of entry onto the site which everyone (including the researchers) is required to abide by.

23. Questions

Any questions regarding the project can be directed to the **Principal Investigator**:

Prof Luke Downey: ldowney@swin.edu.au, (03) 9214 5781

Any medical enquiries can be directed to the **Medical Officer:**

Prof Edward Ogden: eogden@swin.edu.au, (03) 9214 5098

24. Complaints or concerns

This project has been approved by or on behalf of Swinburne's Human Research Ethics Committee (SUHREC) in line with the *National Statement on Ethical Conduct in Human Research*. If you have any concerns or complaints about the conduct of this project, you can contact:

Research Ethics Officer, Swinburne Research (H68),
Swinburne University of Technology, PO Box 218, HAWTHORN VIC 3122.
Tel: (03) 9214 5218 - Email: resethics@swin.edu.au

In the unlikely event that the testing procedures used in this study lead to personal distress, we encourage you to contact your treating doctor, Lifeline and/or the Swinburne Psychology Clinic for professional support (details below).

**Swinburne Psychology Clinic**<br>Level 4, The George, Wakefield St, Hawthorn, 3122<br>Open: 9am-5pm weekdays<br>Phone: (03) 9214 8025	**Lifeline**<br>24-hour Crisis Support<br>Phone: 13 11 14

25. Consent Form

Sign the consent form only after you have made up your mind to take part in this clinical trial. All study participants must be provided with a signed and dated copy of the Participant Information and Consent Form for their personal record:

I, ………………………………………………………………… (Name of participant) agree to participate in a research project entitled: **A Closed-Circuit Track Trial to Assess Risk, Impairment and Performance from Medical Cannabis (CANTRACK).**

I consent to participate in the study named above. I have been provided with a copy of the study information statement and this consent form, and any questions I have asked have been answered to my satisfaction.

By signing this consent form you agree to:

- Be interviewed by the investigator
- Truthfully answer all questions about your medical history and medical cannabis use
- Attend Swinburne University in Hawthorn, METEC Driver Training in Bayswater North and AARC in Wensleydale on the specific dates/times agreed with the researchers

Your agreement is based on the understanding that:

- You may withdraw at any time.
- You freely agree to participate in this project according to the conditions on the Participant Information.
- You will be given a copy of the Participant Information and Consent Form to keep.
- You have received a verbal explanation of the study, including its procedure and risks
- Your consent to participate in this project is given freely.
- You acknowledge that any personal or health information about me which is gathered during your participation in this study will be collected, retained, accessed, and analysed by the researcher(s) for the purpose of this research project, as well as future, related, research projects. In the present analysis and any future analyses, you acknowledge that your identity will not be disclosed.

- No raw data, video or potentially identifiable data will be made publicly available or shared with any other person or organisation not listed in this application. In the case that a short video segment was to be made available to media or promotional videos, explicit and separate written and informed consent will be obtained by the research team.
- The researcher has agreed not to reveal my identity and personal details if information about the project is published or presented in any public form. I understand the time involved in each of the testing sessions.
- You understand that participation in this study does not exempt you from potential charges associated with testing positive to cannabis if I were to be subject to a roadside drug test after leaving the trial site.

I agree to my de-identified data being used for future research projects conducted by the same team or by other researchers (please tick one):

☐ **Yes**

☐ **No**

Name of Participant ....

Signature ....**Date** ....

Declaration by Principal Investigator, Co-Investigator (CI) or Research Assistant (RA)
A verbal explanation of the research project, its procedures and risks has been given to the participant and I believe that the participant has understood that explanation.

Name of Investigator†..

Signature ....**Date** ....

† A senior member
